# Magnetic resonance imaging and clinical findings in seminal vesicle pathologies

**DOI:** 10.1590/S1677-5538.IBJU.2017.0153

**Published:** 2018

**Authors:** Zafer Ozmen, Fatma Aktas, Nihat Uluocak, Eda Albayrak, Ayşegül Altunkaş, Fatih Çelikyay

**Affiliations:** 1 Department of Radiology, School of Medicine, Gaziosmanpasa University, Tokat, Turkey; 2 Department of Urology, School of Medicine, Gaziosmanpasa University, Tokat, Turkey

**Keywords:** Seminal Vesicles, Pathology, Magnetic Resonance Imaging

## Abstract

**Purpose:**

Congenital and acquired pathologies of the seminal vesicles (SV) are rare diseases. The diagnosis of SV anomalies is frequently delayed or wrong due to the rarity of these diseases and the lack of adequate evaluation of SV pathology. For this reason, we aimed to comprehensively evaluate SV pathologies and accompanying genitourinary system abnormalities.

**Materials and Methods:**

Between March 2012 and December 2015, 1455 male patients with different provisional diagnosis underwent MRI. Congenital and acquired pathology of the SV was identified in 42 of these patients. The patients were categorized according to their SV pathologies. The patients were analyzed in terms of genitourinary system findings associated with SV pathologies.

**Results:**

SV pathologies were accompanied by other genitourinary system findings. Congenital SV pathologies were bilateral or predominantly in the left SV. Patients with bilateral SV hypoplasia were diagnosed at an earlier age compared to patients with unilateral SV agenesis. There was a significant association between abnormal signal intensity in the SV and benign prostate hypertrophy (BPH) and patient age.

**Conclusion:**

SV pathologies are rare diseases of the genitourinary system. The association between seminal vesicle pathology and other genitourinary system diseases requires complete genitourinary system evaluation that includes the seminal vesicles.

## INTRODUCTION

The seminal vesicles (SV) are part of the male reproductive urogenital organs. The development of the SV is closely linked to ureter and kidney development. SV agenesis is the most common congenital SV pathology that in rare cases may lead to infertility. In addition to congenital anomalies, nonspecific inflammation of SV can also lead to SV hemorrhages. Diseases originating from the prostate, bladder, and rectum can affect the SV (1, 2). The diagnosis of SV anomalies is frequently delayed or inaccurate due to the infrequency of SV dysfunction and the lack of awareness. Genitourinary system abnormalities can be accompanied by SV pathologies. Therefore, a full diagnosis should include evaluation of the SV.

A remarkable advance in cross-sectional imaging has resulted in increased detection of SV pathology (3). SV pathology is generally evaluated through magnetic resonance imaging (MRI). However, multi-detector computed tomography (MDCT) and ultrasonography (US) are also useful diagnostic tools for evaluating the SV.

The aim of this study is to evaluate the prevalence of congenital and acquired SV pathology, and secondly to define accompanying genitourinary system abnormalities from a comprehensive point of view.

## MATERIALS AND METHODS

### Patients

This study was reviewed and approved by the Institutional Review Board of our hospital. Informed consent was waived, since the study was retrospective and personal health information was not disclosed.

The medical records of 1455 male patients that were referred for MRI analysis to the Gaziosmanpaşa University, School of Medicine, Department of Radiology between March 2012 and December 2015, were evaluated. Complete MRI findings were analyzed and the data was retrieved from the hospital information system. MRI examination, age and the clinical center that first diagnosed the patient were recorded.

### MRI protocol

All MRI examinations were performed by using an 8-channel body phased array (PPA) coil with a 1.5 Tesla magnet (GE Signa Excite 14.0, GE Medical Systems, Milwaukee, Wisconsin, USA). The MRI protocol consisted of axial fast spin echo T1-weighted (T1W), fat-suppressed fast spin echo T1W, fast spin echo T2-weighted (T2W), fat-suppressed fast spin echo T2W, coronal fast spin echo T2W, sagittal fast spin echo T2W images and axial plan diffusion-weighted echo planar imaging (DW-EPI) sequence using b=0, b=500, and b=1000s/mm^2^ parameters.

MRI parameters were TR/TE 550/15 (T1W-image), 3625/85 or 4400/85 (T2W-image), 3900/85 (T2W image with fat saturation), 3 or 5mm slice thickness, 1 or 1.5mm intersection gap, 26 or 28cm field of view (FOV), 2-3 excitations (NEX), and a 384×256 or 320×288 matrix. All patients underwent imaging following a minimum fasting period of 8 hours. During the investigation, 10-15mlt gadolinium benzoate derivative intravenous contrast agent (Omniscan; GE Healthcare, Cork-Ireland) was administered at 0.8mlt/sec.

### Analysis of MRI images

Data collection and analysis were performed using an archive and communication system (PACS) workstation (Centricity RA 1000, GE Healthcare Milwaukee, WI, USA). All images were evaluated by a radiologist with at least 10-years of abdominal MRI experience. The radiologist was blinded to the patient's clinical data, official reports, radiological examinations performed outside of the check-up program and the medical records from the referral examination. The radiologist indicated the presence and type of SV anomaly.

The following data was analyzed for all patients: SV presence, size, shape, and signal features, and vas deference (VD) presence, size and shape. Previously reports have shown that a normal SV size is 3.0×0.8cm±0.4, and a normal ampulla of VD is 0.4cm±0.1. Normal SV wall thickness as measured by MRI is reported as 1-2mm (4-7). SV's are classified in three categories i.e. normal, hypoplasia, and agenesis (8). The same classification will be used in this study. In this study, a SV size smaller than previously reported was classified as hypoplasic SV's, whereas absences of SV's were accepted as SV agenesis.

We regarded T1W hyperintensity of the SV as abnormal signal intensity (regardless of whether there were hypointense signals in T2W sequences or not). In order to evaluate abnormal signal intensity of SV's in T1W sequences, we searched for reference structures i.e. subcutaneous or pelvic adipose tissue, or bone marrow on the same sequence. Since adipose tissue produces extremely hyperintense signals, bone marrow was used as reference (9).

All pelvic structures were evaluated in terms of the pelvic pathology accompanying SV pathology. Moreover, the presence, localization and size of the kidneys were also analyzed in patients with SV pathology.

Urinary system ultrasonography was used for evaluation of renal anomalies in patients with SV pathology from whom only lower abdominal MRI analysis was performed. Urinary system ultrasonography was performed with 3.5MHZ PVT-375BT Toshiba Aplio 500 and was analyzed by the same radiologist that assessed the MRI sequences.

### Statistical analysis

All data were evaluated using IBM SPSS Statistics Version 20 software. Count (n) and percentage (%) were used for descriptive statistics. The Fischer Chi-square test was applied for comparisons of grouped categorical variables made. The Mann-Whitney U test was used to evaluate differences in continuous variables. P-value of <0.05 was accepted as statistical significant.

## RESULTS

Congenital and acquired pathologies of the seminal vesicle were identified in 42 patients. The age varied between 21 and 76 years with an average age of 48.7 years. Patients were referred to our department due to the following preliminary diagnoses: infertility (28.5%, n=12), hematospermia (14.2%, n=6), perianal fistula (9.5%, n=4), rectal cancer (9.5%, n=4), pelvic pain (4.8%, n=2) prostate cancer (4.8%, n=2), liver mass (4.8%, n=2), cystitis (4.8%, n=2), adrenal adenoma (4.8%, n=2), perianal abscess (4.8%, n=2), bladder cancer (2.4%, n=1), primary enuresis (2.4%, n=1), renal mass (2.4%, n=1) and hematuria (2.4%, n=1).

**Figure 1:**
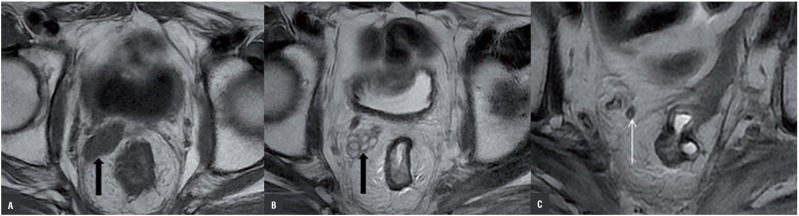
Left SV agenesis of a 63 years old male patient who had colon ca. Axial (a) T1 weighted MR image, axial; (b) T2 weighted MR images show left SV absence. Right SV normal (thick black arrow). Axial (c) T2 weighted MR image show; SV absence was associated with left VD absence. Right VD normal (white thin arrow). Postoperative changes at the anastomosis line of the rectosigmoid junction.

Ten patients presented unilateral SV agenesis and nine patients (90%) had unilateral left SV agenesis (Figure-1). Seventy percent of the patients had unilateral left SV agenesis accompanied by anomalies such as renal agenesis, renal ectopia, VD agenesis or hypoplasia. The genitourinary system findings accompanying to unilateral left SV agenesis are presented in Table-1. Only one patient (10%) had unilateral right SV agenesis, which was observed in combination with abnormal signal intensity in the left SV. Bilateral kidneys and VDs appeared normal.

**Table 1:** Genitourinary system abnormalities accompanying unilateral left SV agenesis.

Findings	%	Patient number (n)
Ipsilateral left renal agenesis	44,5	4
Ipsilateral left VD agenesis	44,5	4
Ipsilateral left ectopic kidney	11,1	1
Left undescended testis	11,1	1
Ipsilateral left VD hypoplasia	11,1	1

**SV** = Seminal vesicle; **VD** = Vas deference

Two patients had bilateral SV agenesis accompanied by bilateral VD agenesis. One patient had a 1cm midline smooth marginated utricle cyst on the prostate gland.

**Figure 2:**
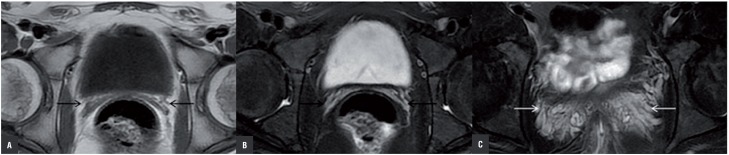
Bilateral SV hypoplasia in an 18 year old male patient with complaints of pelvic pain. Axial (a) T1 weighted MR image and axial (b, c) fat-saturated T2 weighted MR images show both SV were significantly small (black arrow). Congestion were visualized in pelvic vascular structures at the SV location (white arrow).

Ten patients were diagnosed with SV hypoplasia. Of the 10 patients, 8 patients had bilateral SV hypoplasia and 2 patients had unilateral left SV hypoplasia (Figure-2). Isolated hypoplasia of the right SV was not observed in any of the patients. One patient with left SV hypoplasia had also left VD agenesis, whereas the other patient with left SV hypoplasia had contralateral ectopic kidney. The VDs were thinly calibrated in one patient with bilateral SV hypoplasia. Diameter of VD in this patient was measured at 2mm. The age of the patients with bilateral SV hypoplasia was 21-25 years.

A comparison of patients with and without unilateral SV agenesis revealed no significant difference in age distribution (p >0.05). However, a statistically significant difference in age was found between patients with and without bilateral SV hypoplasia (p <0.05) (Table-2). Patients with bilateral SV hypoplasia (26, 28 age) were diagnosed at a significantly younger age as compared to patients with unilateral SV agenesis (p<0.05). However, due to inadequate number of patients with unilateral SV hypoplasia and bilateral SV agenesis, we could not perform statistical analysis for these two groups. All of the patients with bilateral hypoplasia went to the urology outpatient clinic at a younger age complaining of infertility.

**Table 2:** Age distribution in SV pathologies.

SV Pathologies		n	Median (Q1-Q3)	p
Bilateral SV hypoplasia	(+)	8	23,5 (22,25-24,75)	<0,001
	(-)	34	61,0(45,5-68,0)	
Unilateral SV agenesis	(+)	10	57,0 (40,0-62,0)	0,951
	(-)	32	53,0 (25,0-68,0)	

**SV** = Seminal vesicle

**Figure 3:**
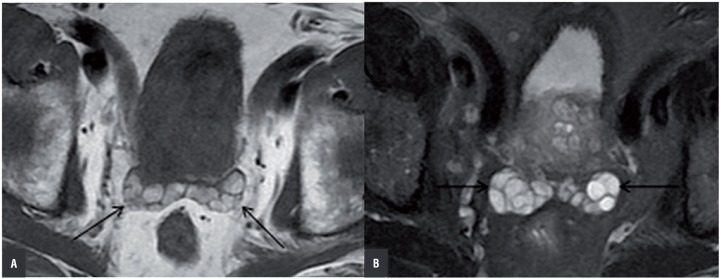
Bilateral SV hemorrhage in a 75 year old patient with BPH. Axial (a) T1 weighted MR image, axial (b) fat-saturated T2 weighted MR image show; hyperintense signal features on both SVs in T1 and T2 weighted series (black arrows).

SV abnormal signal intensities (ASI) were noted in 17 patients. Bilateral ASI was found in 10 of the 17 patients (Figure-3). One patient had right ASI and 6 patients had left ASI. Left ASI was significantly more common than right ASI, which was similar to our observations regarding unilateral SV agenesis and unilateral SV hypoplasia. ASI was defined as profoundly hyperintense signals in T1W sequences and mild hyperintense signals in T2W sequences of SV. Left-side preponderance in patients with agenesis, hypoplasia and ASI of the SV is shown in Table-3. Nine cases (52.9%) of ASI were accompanied by benign prostate hyperplasia (BPH). In one patient, left ASI was accompanied by contralateral right SV agenesis. One patient with bilateral ASI had the SVs invaded with bladder tumor, and marked dilatation of both SVs in addition to the ASI. Another patient with left ASI had postoperative recurrence of rectal cancer and invasion of the left SV. The VDs of all patients with ASI were normal. ASI disappeared following antibiotic and anti-inflammatory treatment in three patients. However, twelve patients did not show changes in their ASI's at follow-up examinations. None of the patients had an underlying pathology such as neoplasia, vascular malformation, or cyst. The patients with bladder tumor and rectal tumor had no change in ASI at the follow-up examinations.

**Table 3:** Left side preponderance in SV pathologies.

SV Pathologies	Right	Left	% Right	% Left
Unilateral SV agenesis	1	9	10	90
Unilateral SV hypoplasia	0	2	0	100
SV abnormal signal intensities	1	6	14,2	85,8

**SV** = Seminal vesicle

There was a significant difference in age between patients with and without ASI (p<0.05) (Table-4). Furthermore, we found that ASI was more prevalent among patients with BPH (p<0.05). The relationship between ASI and BPH is shown in Table-5.

**Table 4:** Association between SV abnormal signal intensity and patient age.

	n	Median (Q1-Q3)	p
SV abnormal signal intensities (+)	17	65,0 (52,5-69,0)	<0,001
SV abnormal signal intensities (-)	25	38,0 (24,0-61,0)	

**SV** = Seminal vesicle

**Table 5:** Association between SV abnormal signal intensity and BPH.

	BPH (+)		BPH (-)		Totally		p
	n	%	n	%	n	%	
SV abnormal signal intensities (+)	9	52,9	8	47,1	17	100	<0,001
SV abnormal signal intensities (-)	1	4,8	24	95,2	25	100	

**BPH** = Benign prostate hyperplasia; **SV** = Seminal vesicle

SV cysts were present in 2 patients. In one patient, several adjacent thin-walled cysts on the left SV with the largest being 14×20mm in size was observed. In the second patient, the size of the SV increased with bilateral adjacent cysts. The largest cyst in the second patient had a size of 31mm and was presented in both SVs. The VDs and the kidneys were apparently normal. One patient had - vesiculitis, which showed diffuse and marked thickening in the bilateral seminal vesicle walls (3mm), with septal and peripheral contrasting of the wall. Seminal vesiculitis was accompanied by ASI in the left SV. Following antibiotic treatment, both the thickening and contrast uptake at the wall of SV and the ASI disappeared. In one patient, a region of proximal 7mm on the right SV was observed which corresponded to a hypointense signal in T1W and T2W sequences and no contrast uptake. This region was interpreted as calcification and confirmed by CT examinations.

All SV pathologies detected in our study group are shown in Table-6. Twenty-four patients (57.1%) had congenital SV pathology and 18 patients (42.9%) had acquired pathologies of the SV. All SV pathologies were accompanied by various genitourinary system abnormalities (Table-7).

**Table 6:** Congenital and acquired SV pathologies.

SV Pathologies	%	Patient number (n)
SV abnormal signal intensities	40,5	17
Unilateral SV agenesis	23,8	10
Bilateral SV hipoplasia	19	8
Bilateral SV agenesis	4,8	2
Unilateral SV hypoplasia	4,8	2
SV cyst	4,8	2
Seminal vesiculitis	2,4	1
SV calcification	2,4	1

**SV** = Seminal vesicle

**Table 7:** Additional findings in patients with congenital and acquired SV pathologies.

		%	Patient number (n)
**Additional findings with congenital SV pathologies**		57,1	24
	Ipsilateral VD agenesis	11,9	5
	Ipsilateral renal agenesis	9,5	4
	Bilateral VD agenesis	4,8	2
	Bilateral VD hypoplasia	2,4	1
	Ipsilateral VD hypoplasia	2,4	1
	Ipsilateral ectopic kidney	2,4	1
	Contralateral pelvic kidney	2,4	1
	Ipsilateral undescended testis	2,4	1
	Utricle cyst	2,4	1
**Additional findings with acquired SV pathologies**		**42,9**	**18**
	BPH	50	9
	Contralateral ectopic kidney	5,5	1
	SV dilatation	5,5	1

**SV** = Seminal vesicle

**VD** = Vas deference

**BPH** = Benign prostate hyperplasia

## DISCUSSION

One of the most common congenital abnormalities of the SV is agenesis. There are two types of SV agenesis i.e. unilateral and bilateral SV. Unilateral agenesis of seminal vesicle was quite rare and its incidence is between 0.61% (10). Unilateral SV agenesis is the result of an embryological trauma occurring before 7th week of gestation, when the ureteric bud develops from the mesonephric duct. Unilateral SV agenesis is associated with ipsilateral or/ and bilateral agenesis or ectopy of the vas deferens (10-13). Unilateral SV agenesis is also associated with ipsilateral renal agenesis (79%) or renal anomalies (12%). However, unilateral SV agenesis may occur in the presence of normal kidneys (9%). Sixty-four to seventy three percent of bilateral SV agenesis is related with cystic fibrosis transmembrane conductance regulator gene CFTR gene mutation. The incidence of bilateral SV agenesis is not known clearly (10). Bilateral SV agenesis occurs due to development of luminal obstruction possible resulting from dark secretion. Bilateral SV agenesis is associated with VD agenesis. The kidneys are generally normal in these cases (3, 4).

In this study, the incidence of unilateral SV agenesis was 0.69%, which was in accordance with the literature. The incidence of bilateral SV agenesis was 0.14%. Unilateral SV agenesis was accompanied by renal agenesis, renal ectopy, VD agenesis and VD hypoplasia. Concurrence of ipsilateral renal agenesis with unilateral SV agenesis (44.5%) was less common in this study than previously reported. The prevalence of other accompanying abnormalities was not significantly different than previously reported. Similar bilateral SV agenesis was accompanied by VD agenesis as previously reported (3, 4).

SV hypoplasia is a congenital deformation that results in a smaller than normal SV. The SV size typically increases with age before decreasing in advanced age (4). The size of SV generally decreases after the age of 70 (10). SV hypoplasia is generally observed as bilateral. In our study, most of the SV hypoplasia was bilateral (80%) or on the left side (20%). Accompanying VD and renal anomalies were on the left side. Additionally, we found that left SV hypoplasia was accompanied by right pelvic kidney in one patient. SV pathologies are mostly accompanied by ipsilateral renal anomalies, and the embryologic mechanisms of these anomalies are well defined (13, 14). However, contralateral renal anomalies accompanying SV pathologies are rare in the literature (12), and there is no convincing explanation about the embryological mechanism leading to this concurrency. Therefore, we reason that this type of concurrence was incidental.

The seminal fluid contains semenogelin, proteins, enzymes, fructose, vitamin C and other enzymes that provide nutrition for the spermatozoa (9). Due to this heterogeneous nature, the seminal vesicles can sometimes show hyperintense signal features on T1W images that resemble hemorrhage. In patients with hematospermia, the content of the seminal vesicles is hemorrhagic. Hematospermia is generally associated with nonspecific inflammation of the prostate or SV (9). Infection/inflammation, neoplasia, vascular disease, trauma and cysts are the most common causes of SV hemorrhage. A prior study speculated that impingement and distension might develop in the ejaculate canal due to stasis, BPH and age, leading to hemorrhage and increased pressure in the SV. Hematospermia is most commonly diagnosed in the 5^th^ and 6^th^ decades (15). We found a strong correlation between BPH and ASI (p <0.001) and between advanced patient age and ASI (p<0.05). Interestingly, 35.3% of patients with ASI had hematospermia. The strong relationship between ASI, age and BPH, let to the reasoning that abnormal signal intensity in majority of our patients was related to the increased pressure inside SV. We hypothesized that the reason for ASI among these patients was due to sub-acute hemorrhage. However, we could not evaluate this relationship since seminal fluids are not routinely collected in patients without hematospermia. In most of our patients, changes in the signal intensity at follow-up examinations were not found. In three patients ASI returned to normal after antibiotic and anti-inflammatory treatment. The cause of ASI in these three patients was therefore considered to be related to infection/inflammation.

As with unilateral SV agenesis and unilateral SV hypoplasia, unilateral abnormal signal intensity in the SV had a high left-side preponderance. Left SV pathology was present in 91.6% of our patients with unilateral SV pathologies. To our knowledge, there is no information in the literature regarding left side preponderance of SV pathologies. For congenital SV anomalies, embryological stages in the development of SV's may be responsible for the left side preponderance. However, no embryological mechanism leading to higher prevalence of left SV pathologies has been reported. Interestingly, acquired SV anomalies also have left-side preponderance, which is quite puzzling. As a result of these findings, further investigation of left side dominance of SV abnormalities is needed.

Congenital SV cysts may occur as an isolated finding but are most commonly associated with ipsilateral renal agenesis or dysgenesis (16). This study also showed SV cysts but we did not detect any accompanying genitourinary system abnormalities.

There are several important limitations to our study. First, the numbers of patients with congenital and acquired pathologies of SV was low despite the large screening population. Therefore, preponderance of left sided SV agenesis may be a consequence of the limited number of patients in our study. Thus, studies with a larger number of SV abnormalities are necessary to demonstrate left-side preponderance. Secondly, this study is retrospective which did not allow us to perform puncture or biopsy to determine whether abnormal signal intensity observed at T1W images was due to hemorrhagic seminal fluid.

In conclusion, pathologies of the SV are rare. Although mechanisms of SV pathologies and accompanying anomalies have been well defined, we may encounter some pathology that cannot fully explain these mechanisms. SV pathologies are accompanied by genitourinary system abnormalities and are most commonly seen on the left side. Larger studies will be required to confirm left side preponderance of SV pathologies. Similar to previous studies, we observed an association between BPH and ASI. Interestingly, bilateral SV hypoplasia was diagnosed at a significantly younger age, whereas no association was found between age and unilateral SV agenesis.
